# Peripubertal lung growth pattern in Japanese school children

**DOI:** 10.14814/phy2.70508

**Published:** 2025-08-26

**Authors:** Satoshi Konno, Masataka Taguri, Hiroshi Odajima, Mihoko Minami, Toru Takebayashi, Hiroshi Nitta, Masaharu Nishimura

**Affiliations:** ^1^ Department of Respiratory Medicine, Faculty of Medicine Hokkaido University Sapporo Japan; ^2^ Department of Health Data Science Tokyo Medical University Shinjuku‐ku Japan; ^3^ National Hospital Organization Fukuoka National Hospital Fukuoka Japan; ^4^ Department of Mathematics, Faculty of Science and Technology Keio University Minato‐ku Japan; ^5^ Department of Preventive Medicine and Public Health Keio University School of Medicine Minato‐ku Japan; ^6^ National Institute for Environmental Studies Tsukuba Japan; ^7^ Hokkaido Medical Institute of Respiratory Diseases Sapporo Japan

**Keywords:** children, lung grwoth, puberty

## Abstract

The peripubertal growth pattern of lung function remains underexplored in relation to height growth. This study aimed to first clarify the relationship between the age at peak growth velocity in lung function variables and the age at peak height velocity (APHV) and second identify sex differences in lung function growth patterns. Lung function and height were measured annually in children aged 9–15 years (elementary schools, *N* = 1307; junior high schools, *N* = 792) from 2011 to 2018. Children were categorized quarterly according to APHV, using the Super Imposition by Translation and Rotation model. The age at peak growth velocity for forced vital capacity (FVC) and forced expiratory volume in 1 s (FEV_1_) lagged behind APHV by 2–12 months. The later the APHV, the greater the numerical lag, although this was not significant. In males, but not females, the trajectory of FEV_1_/FVC values gradually decreased to reach the lowest levels and then gradually increased with age (U‐shaped curve) in all quartiles. Both FVC and FEV_1_ overwhelmed in males compared with those in females when the height exceeded 150–160 cm. Our results highlight significant variability in peripubertal lung growth with height and sex‐related differences in the growth of airways and parenchymal components.

## 1 INTRODUCTION

The growth pattern of lung function in childhood may affect finally attained lung function variables in adults, which in turn may be linked with the future development of chronic pulmonary diseases such as asthma and chronic obstructive pulmonary diseases (COPD) (Bui et al., 2017; Bui et al., 2018; Dharmage et al., 2023; Postma et al., 2015). Thus, accurate and precise evaluations of the growth pattern of lung function in children have garnered renewed attention. Studies that were exclusively conducted in Western countries focused on the peripubertal lung growth pattern and retrospectively calculated the average value of pulmonary function test (PFT) indices in each age group to depict the pulmonary‐function growth pattern (Borsboom et al., 1996; Lebowitz & Sherrill, 1995; Sherrill et al., 1989; Wang et al., 1993). To our knowledge, no study has involved prospective, repeated lung‐function measurements using the same equipment for the same individuals across the study period.

It is widely recognized that the age of puberty varies among children, complicating the standardization of growth depictions, including height, for any organ (Tanaka et al., 1998; Yousefi et al., 2013). Earlier pubertal development is associated with reduced lung growth later in life as well as with potential future risk of pulmonary diseases in young adults (Castro‐Rodríguez et al., 2001; Guerra et al., 2004; Macsali et al., 2011; Salam et al., 2006; Varraso et al., 2005). Recently, Mahmoud et al. (2018) analyzed the velocity of height growth by differentiating the longitudinal height growth trajectories from age 7 to 24 years and derived the values of height growth velocity. The age at which the highest height growth velocity occurred during the observation period was considered the age at peak height velocity (APHV). The authors demonstrated that APHV is cross‐related to self‐reported pubertal timing and is significantly associated with the maximum lung function attained at the age of 24 years in both sexes. Specifically, a later APHV is associated with higher lung function in adults (Mahmoud et al., 2018). These results indicate that APHV is a good parameter for predicting puberty and estimating future lung function. Wang et al. (1993) showed that lung growth lagged behind height and that the earlier the onset of the height growth spurt, the greater the lag between height and lung function growth. However, they only examined individuals in whom apparent peak lung growth could be identified during the study period, thus limiting the study sample.

The geometry of airways and lung parenchyma is known to be affected both by genetic and environmental factors. Green et al. and Mead first proposed the term “dysanapsis,” which indicates a disproportionate growth of airways and lung parenchyma (Martin et al., 1987; Mead, 1980; Nishimura et al., 1991). Recently, this concept has been implicated in the pathogenesis of COPD, which is defined by a reduced ratio of the forced expiratory volume in 1 s (FEV_1_) to forced vital capacity (FVC) of the lungs (FEV_1_/FVC), specifically an FEV1/FVC ratio of <70% (McGinn et al., 2023; Smith et al., 2020). Importantly, low FEV_1_/FVC in boys, compared with that in girls, may account for the higher susceptibility of boys to asthma (Akinbami et al., 2012; Ito et al., 2023; Leynaert et al., 2012) and/or passive smoking (Lebowitz et al., 1992; Murray & Morrison, 1989). This phenomenon could also explain the prevalence and severity of cystic fibrosis (Harness‐Brumley et al., 2014) and the sex differences in elite swimming performance in youths (Senefeld et al., 2019).

The Ministry of the Environment in Japan monitored the concentrations of particulate matter with diameter ≤2.5 μm (PM2.5) in various regions of the country and prospectively and repeatedly measured lung function in children in each region from 2011 (2012) to 2017 (2018). They concluded that PM2.5 levels in Japan did not affect lung growth in children from 9 to 12 years of age (Takebayashi et al., 2022). In this study, PFTs were conducted. Subsequently, we extended this study for approximately 60% of the aforementioned study population until they reached the age of 15 years.

Utilizing this highly valuable dataset, we attempted to examine lung growth pattern around puberty in Japanese children. The present study has the following two objectives:

1. To depict the trajectory of lung function growth in relation to APHV. To this end, we categorized all study participants into quartiles according to APHV and compared the lag of peak growth between height and lung function variables in each group.
2. To depict and compare between male and female children the trajectory of FEV_1_/FVC around puberty, a parameter representing dysanapsis.

This study was conducted to offer clinicians and researchers new insights into the interpretation of peripubertal lung function measurements, potentially extending to the remainder of adolescence.

## 2 METHODS

Details of the materials and methods used in this study have been described in our previous report (Takebayashi et al., 2022); and Appendix S1.

### 2.1 Study design and participants

**FIGURE 1:**
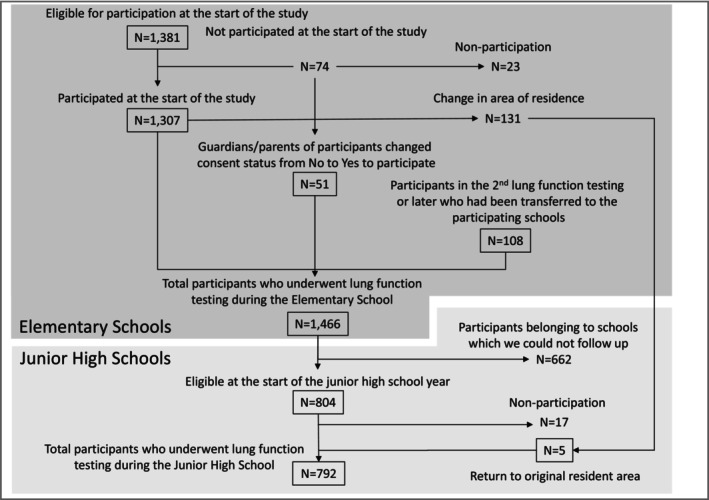
Flowchart for the recruitment and analysis of children in this prospective study.

Figure 1 depicts the flowchart for the recruitment and analysis of participants in this study. We initiated a prospective study for third‐grade elementary school children aged 8–9 years. These children were recruited from nine public elementary schools across six cities in 2011 and seven public elementary schools across four cities in 2012. In total, the study included 16 school communities across 10 cities, from 2011 to 2012, covering a broad geographic area of Japan to capture regional variations in PM2.5 concentrations and represent the nationwide population (Takebayashi et al., 2022).

Follow‐up was conducted almost annually until the children were in the fifth grade (aged 10–11 years) and twice when they were in the sixth grade (aged 12 years). Junior high schools in two cities did not approve of further follow‐up, and this resulted in a reduction in the number of participants (Figure 1). The city altitude in this study ranged from 5 to 592 m. Written informed consent was obtained from the parents of all participants. From elementary schools, 1466 children participated in total, of whom 1307 participated from the beginning. From junior high schools, 792 children participated in total, of whom 723 participated from the first year. In summary, PFTs were conducted in 802 participants (at 11 schools) from 2011 to 2017 and in 664 participants (at 8 schools) from 2012 to 2018. The study was centrally approved by the Ethical Committee of the Ministry of the Environment of Japan (Approval number: 11021001) and also by each regional study center.

### 2.2 Pulmonary function tests

To address the high variability in PFTs among children, we ensured careful administration of PFTs for school children. The detailed methodology for PFTs has been described previously (Takebayashi et al., 2022) and is also provided in Appendix S1.

PFTs for the children in the third to sixth grades were scheduled during the same season every year to minimize seasonal effects such as those of temperature and pollution levels; the same spirometers with Lilly‐type pneumotach sensors (Chest HI801, CHEST M.I., Inc., Tokyo, Japan) were used for testing at all locations. In all schools except two, sixth‐grade children underwent an additional PFT in February or March (i.e., a month before they moved to junior high school). The tests were conducted by trained technicians following the testing protocol of the ATS/ERS standards (Miller et al., 2005). The FEV_1_, FVC, and maximal expiratory flow rate at 50% of FVC (V50) were determined from three satisfactory blows delivered under the guidance of two pediatric pulmonologists.

### 2.3 Data collection

The same equipment was used for all respiratory function measurements, and the measurements were performed carefully by the same group of technicians at the same time of day and year. All students attending regular schools were included in this study. On the day of PFTs, if children were found to have obvious wheezing and/or flu symptoms by the examining physician, their data were excluded from the analyses. We strictly adhered to the ATS/ERS criteria (Miller et al., 2005) for both measurements and the adoption of the best data, which is crucial for children who have difficulty in undergoing PFT. By adopting sophisticated statistical analyses, we could successfully use all the available data and provide an accurate and reliable depiction of lung function growth patterns around puberty, despite the relatively small sample size. Regarding PFT, 244 participants underwent testing four times; 387, five times; 263, six times; and 312, seven times. All raw data are managed by the Ministry of the Environment of Japan.

### 2.4 Statistical analysis

We first estimated APHV for the entire study population using the Super Imposition by Translation and Rotation (SITAR) model (Cole et al., 2010) for fitting height growth curves by sex. The SITAR model is a validated nonlinear mixed‐effects model, and it can estimate individual growth curves by modeling an average curve using fixed effects plus a set of three random effects for each individual that define how his/her growth curve differs from the average. This model provides valid results under the assumption of missing at random. Using the estimated APHV quartiles, we classified the data into four subgroups for each sex. To estimate the mean growth curve of height and lung function indicators (FVC, FEV_1_, V50, FEV_1_/FVC, and V50/FVC) for each quartile group by sex, we used a penalized spline regression model (Wood, 2017) using age as a covariate and height or each lung function indicator as an outcome. Similar models were fitted using height as a covariate. Based on the estimated mean growth curves, we also estimated growth speed curves for each lung function indicator.

We estimated age at peak growth and the corresponding 95% confidence intervals for each lung function variable for all combinations of APHV quartiles and sex. Due to the limited observation period, the peak was identified using the following procedure: (i) From the estimated growth curve, we identified the peak age (age at which the growth speed was maximized). (ii) To confirm the presence of a peak, we examined whether the peak age fell within the 10th and 90th percentiles of the age distribution at the time of measurement. The peak age being within this range indicated the presence of a discernible peak. (iii) To confirm whether the growth speed at peak age was statistically different from those at the 10th and 90th percentiles, we calculated the differences in growth speeds between the peak age and the ages corresponding to the 10th and 90th percentiles in cases where the peak age fell between the 10th and 90th percentiles. We then computed the 95% confidence intervals for the estimated growth speed differences using the bootstrap percentile method (Efron & Tibshirani, 1994). (iv) If the lower limit of the confidence intervals were greater than zero, it indicated a peak in the data. Additionally, we estimated the APHV differences and associated 95% confidence intervals for each lung function variable and height to quantify how much the peak growth of lung function was delayed compared to height. The continuous data in Table 1 showed no significant skewness; therefore, we reported the mean and standard deviation. However, normality tests were not performed, as small deviations from normality can become statistically significant with large sample sizes. All analyses were conducted using R software (version 4.2.0, R Foundation for Statistical Computing, Vienna, Austria), and a two‐sided *p* value of <0.05 was set as the threshold for statistical significance.

**TABLE 1:** Participant characteristics by school grades.

Sex	School	Grade	Number of participants	Age (year) [mean (SD)]	Height (cm) [mean (SD)]	Weight (kg) [mean (SD)]	FVC (L) [mean (SD)]	FEV_1_ (L) [mean (SD)]	V_50_ (L/s) [mean (SD)]	FEV_1_/FVC (%) [mean (SD)]	V_50_/FVC [mean (SD)]
Male	Elementary school	3rd	609	8.82 (0.315)	129.9 (5.61)	27.5 (5.53)	1.92 (0.269)	1.69 (0.233)	2.39 (0.544)	88.2 (4.71)	1.25 (0.275)
		4th	669	9.80 (0.324)	135.2 (6.04)	30.9 (6.46)	2.11 (0.310)	1.85 (0.265)	2.55 (0.588)	87.8 (4.89)	1.22 (0.270)
		5th	671	10.80 (0.326)	141.0 (6.50)	34.6 (7.52)	2.36 (0.353)	2.04 (0.297)	2.77 (0.633)	86.9 (4.83)	1.18 (0.264)
		6th (1)	653	11.79 (0.329)	147.5 (7.59)	39.1 (8.58)	2.65 (0.451)	2.30 (0.383)	3.08 (0.733)	87.0 (5.00)	1.17 (0.260)
		6th (2)	587	12.38 (0.295)	151.7 (7.95)	42.4 (8.98)	2.91 (0.518)	2.52 (0.458)	3.34 (0.858)	86.8 (5.07)	1.16 (0.264)
	Junior high school	8th	372	13.84 (0.363)	162.1 (7.18)	50.5 (9.61)	3.55 (0.596)	3.14 (0.551)	4.14 (1.052)	88.3 (5.64)	1.17 (0.265)
		9th	372	14.83 (0.363)	166.6 (6.05)	54.7 (9.01)	3.93 (0.575)	3.47 (0.531)	4.55 (1.057)	88.6 (5.60)	1.16 (0.249)
Female	Elementary school	3rd	582	8.82 (0.324)	129.1 (5.62)	26.9 (5.11)	1.74 (0.249)	1.58 (0.223)	2.44 (0.534)	90.7 (4.56)	1.41 (0.304)
		4th	652	9.81 (0.330)	135.3 (6.45)	30.4 (5.96)	1.95 (0.288)	1.76 (0.261)	2.69 (0.594)	90.5 (4.37)	1.39 (0.300)
		5th	648	10.81 (0.331)	142.3 (7.09)	34.8 (7.14)	2.23 (0.360)	2.01 (0.328)	3.03 (0.740)	90.1 (4.56)	1.37 (0.305)
		6th (1)	628	11.80 (0.336)	148.6 (6.81)	39.7 (7.93)	2.50 (0.404)	2.27 (0.368)	3.43 (0.780)	90.7 (4.46)	1.38 (0.288)
		6th (2)	536	12.39 (0.292)	151.7 (6.10)	42.9 (7.97)	2.70 (0.417)	2.44 (0.379)	3.61 (0.827)	90.3 (4.83)	1.35 (0.295)
	Junior high school	8th	366	13.87 (0.375)	155.7 (5.10)	48.0 (8.24)	2.96 (0.395)	2.69 (0.352)	3.91 (0.818)	90.9 (4.85)	1.34 (0.289)
		9th	361	14.85 (0.366)	156.9 (5.11)	50.3 (8.04)	3.04 (0.410)	2.75 (0.375)	4.02 (0.853)	90.7 (4.94)	1.34 (0.292)

*Note*: Pupils in the sixth grade were given an additional pulmonary function test in February or March (i.e., a month before they graduated to junior high school). FEV_1_/FVC and V_50_/FVC is the average of the results obtained by calculating the ratio for each individual pulmonary function test.

Abbreviations: FEV_1_, forced expiratory volume in the first 1 s; FVC, forced vital capacity; SD, standard deviation; V50, maximal expiratory flow rate at 50% of FVC.

## 3 RESULTS

**FIGURE 2:**
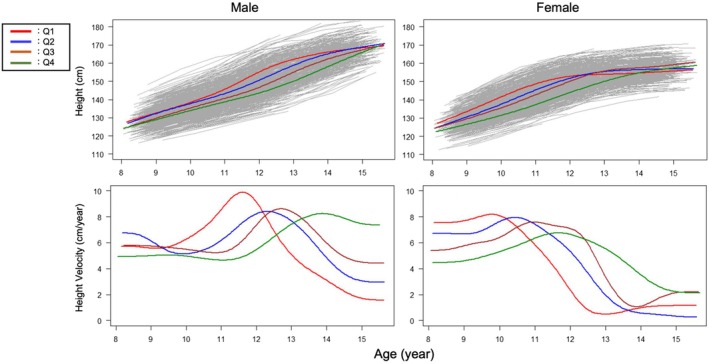
Growth curve and velocity for height in Q1–Q4, which represent quartiles based on the age at peak height velocity (APHV). The left panels are for male children, and the right panels are for female children.

**FIGURE 3:**
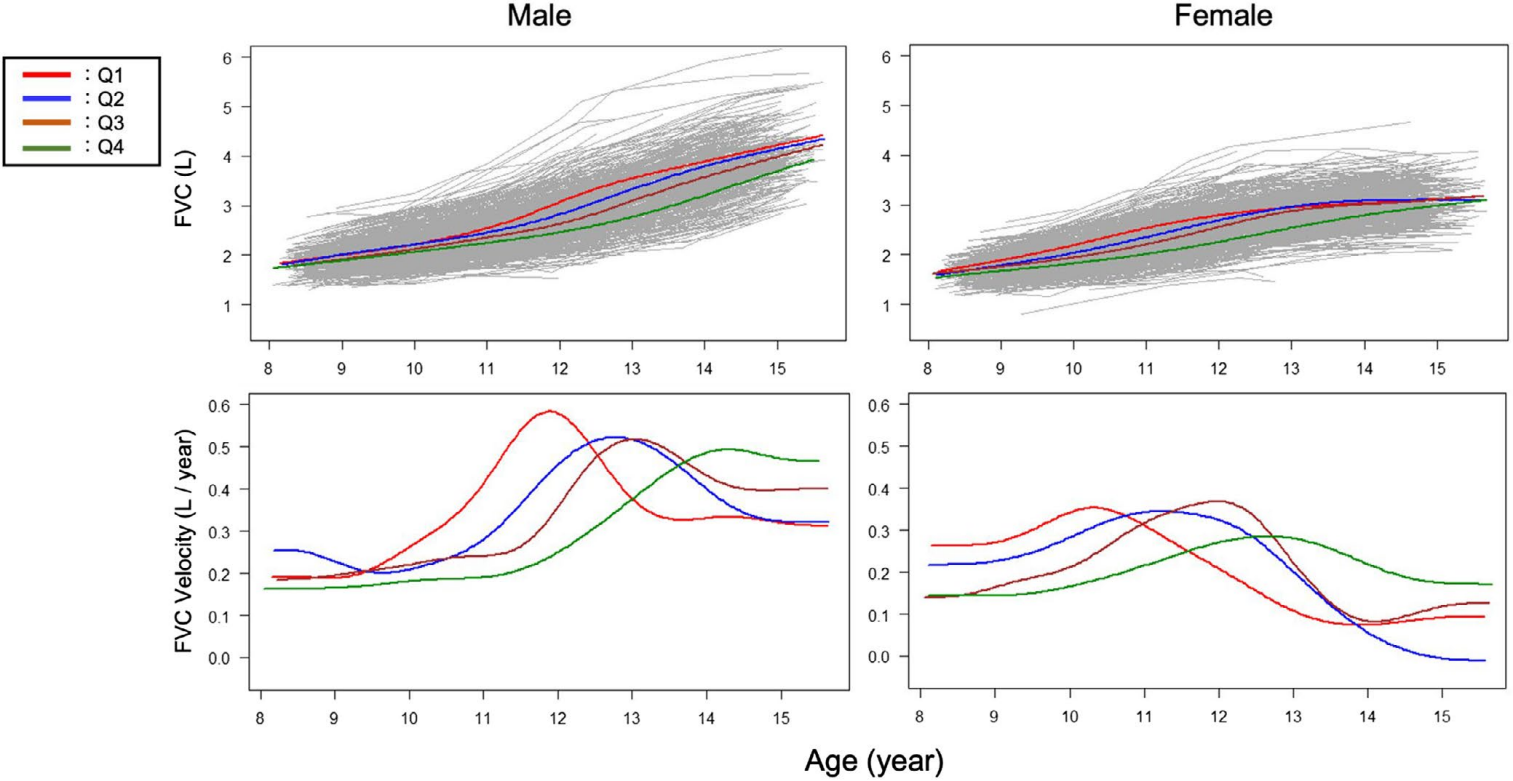
Growth curve and velocity for FVC in Q1–Q4, which represent quartiles based on the age at peak height velocity (APHV). The left panels are for male children, and the right panels are for female children. FVC, forced vital capacity.

**FIGURE 4:**
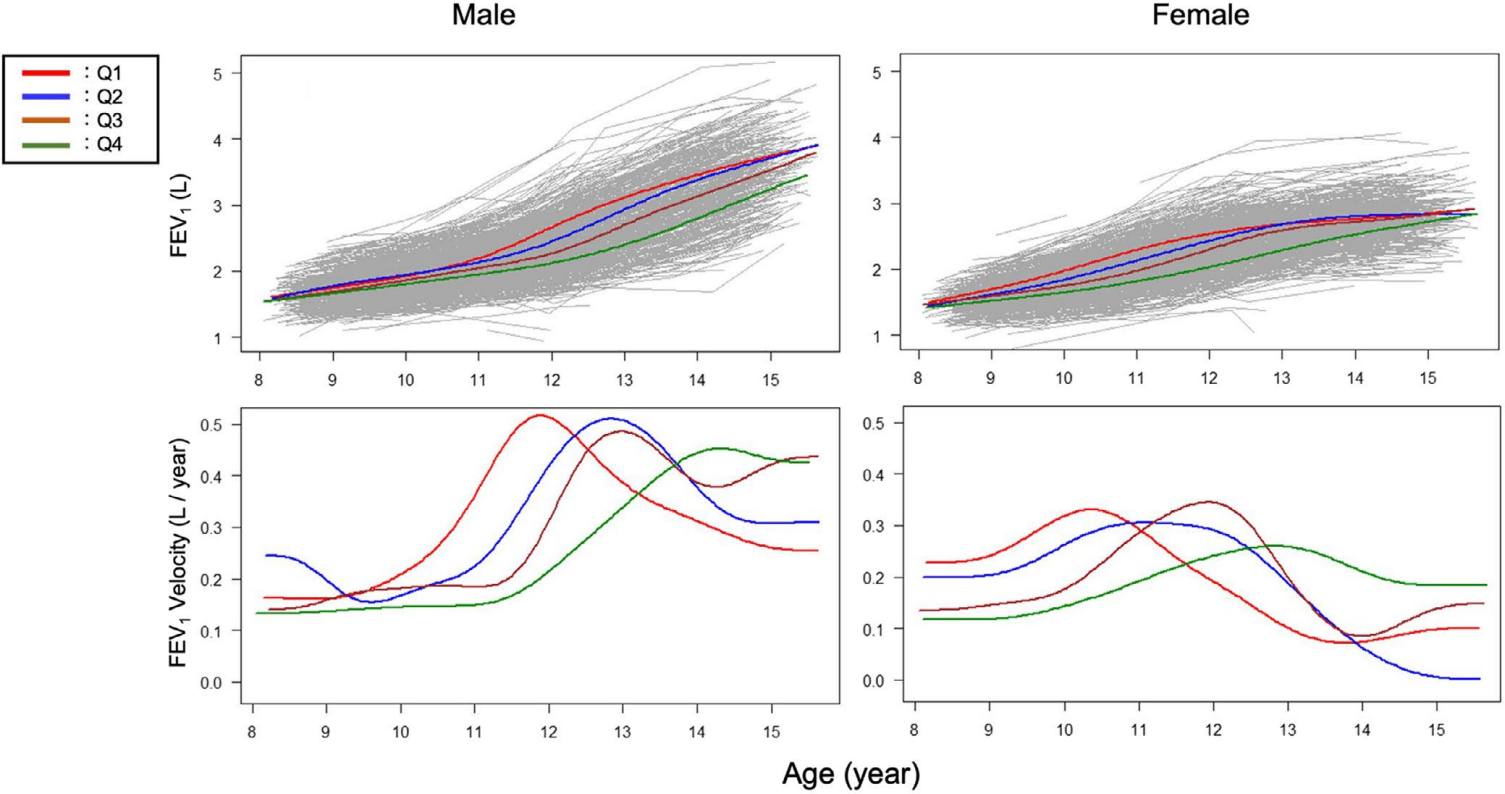
Growth curve and velocity for FEV_1_ in Q1–Q4, which represent quartiles based on the age at peak height velocity (APHV). The left panels are for male children, and the right panels are for female children. FEV_1_, forced expiratory volume in the first 1 s.

Table 1 shows the baseline characteristics of the participants stratified by sex in each grade, and Table S1 shows the results of between‐group comparisons (participants followed up till elementary school level, *N* = 674; participants followed up till junior high school level, *N* = 792). Figure S1 shows the distribution of APHV by sex. The mean APHV was 12.73 ± 0.83 years in male children and 10.90 ± 0.82 years in female children. Considering the significant variation in APHV, we then classified all participants into quartiles. In male children, the boundaries of the quartiles were 12.17, 12.73, and 13.29 years; in female children, they were 10.37, 10.86, and 11.42 years. Figures 2, 3, 4 and Figure S2 show the longitudinal growth pattern and the growth velocity of height, FVC, FEV_1_, and V50 in each group, respectively. The peak of growth velocity in some groups did not appear to be visually identifiable.

Figures S3 and S4 show the growth speed for height, FVC, FEV_1_, and V50 (Figure S2) for male and female children, respectively, in each group. Values shown in the upper left graph denote the ages at peak velocity. In all four groups, growth in each lung function variable significantly lagged behind height, followed by FVC and FEV_1_. Figure S5 shows the length of the lag period between APHV and that of each lung function variable. In male children, peak age differences of height with FVC were 0.28 (years, mean) and 0.45 (years, mean) in Q1 and Q2, respectively, whereas differences of height with FEV_1_ were 0.28 (years, mean) in Q1 and 0.52 (years, mean) in Q2. In Q3 and Q4, the peak age differences could not be calculated, as the peak age could not be identified during the study period. In female children, peak age differences of height and FVC were 0.53 (years, mean) in Q1, 0.79 (years, mean) in Q2, 0.99 (years, mean) in Q3, and 0.99 (years, mean) in Q4, whereas age differences of height and FEV_1_ were 0.57 (years, mean), 0.64 (years, mean), 0.95 (years, mean), and 1.14 (years, mean) in Q1, Q2, Q3, and Q4, respectively. Collectively, the later the APHV, the greater the lag between the peak ages of height and lung function numerically, but these were not significant.

**FIGURE 5:**
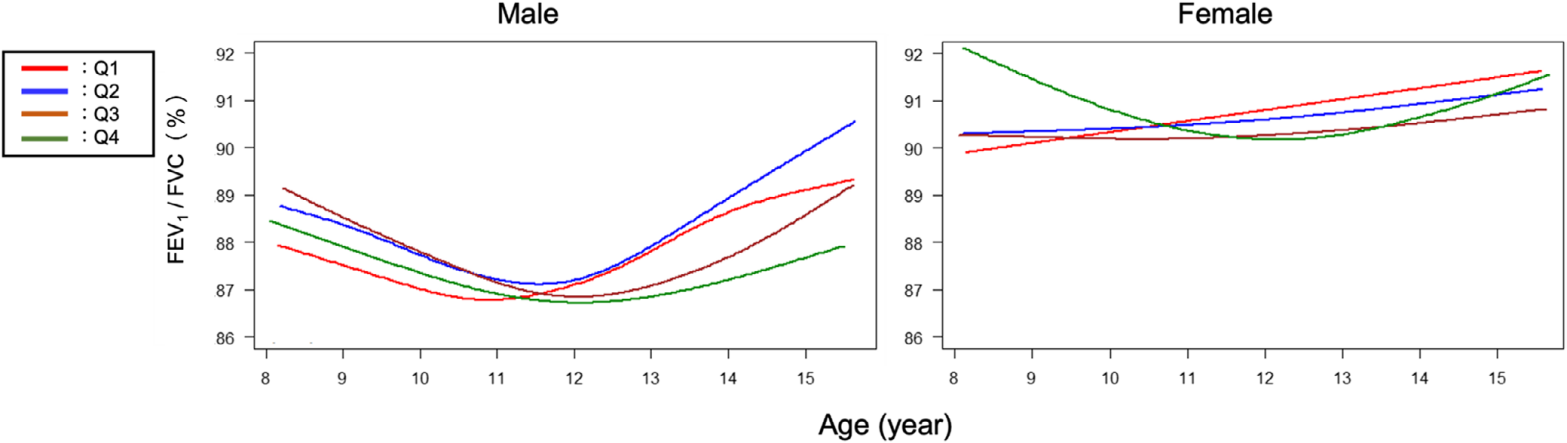
Trajectory curve for FEV1/FVC in Q1–Q4, which represents quartiles based on the age at peak height velocity (APHV). FVC, forced vital capacity; FEV_1_, forced expiratory volume in the first 1 s.

**FIGURE 6:**
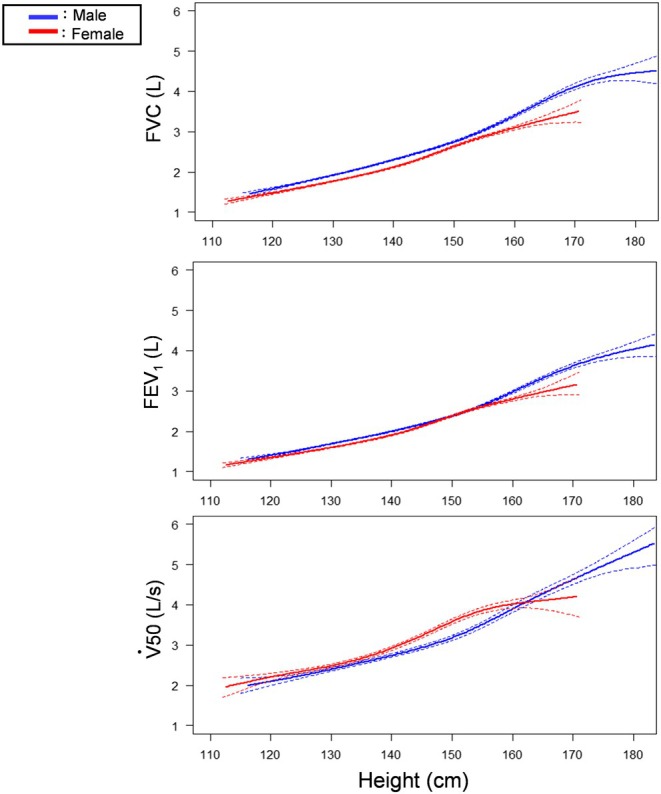
Growth curves of FVC, FEV_1_, and V50 for height in Q1–Q4, which represent quartiles based on the age at peak height velocity (APHV). FVC, forced vital capacity; FEV_1_, forced expiratory volume in the first 1 s.

Figure 5 shows the trajectory of FEV_1_/FVC across the study period. In male children, the trajectory of FEV_1_/FVC values gradually decreased to reach the lowest levels and then gradually increased with age (U‐shaped curve) in all quartiles. It was lowest at the age of 10.88 years in Q1, 11.54 years in Q2, 12.04 years in Q3, and 12.07 years in Q4. In contrast, a U‐shaped pattern was seen in female children only in Q1. Finally, we drew lung function variables as a function of height instead of age (Figure S4), and the four lines were almost identical in both sexes, regardless of quartile. However, the sex‐related difference was evident in absolute values of FEV_1_ as well as FVC when height exceeded 150–160 cm (Figure 6).

## 4 DISCUSSION

In this study, we attempted to depict the lung growth pattern in relation to APHV in children around puberty. Given that the timing of puberty can vary significantly among children, we divided the participants quarterly according to APHV, which is linked with puberty. We then focused on the time lag between the APHV and age at peak lung function growth velocity and found that a later onset of the height growth spurt was associated with a greater lag between the two parameters. Furthermore, we found that the FEV_1_/FVC ratio was consistently lower in males than in females among children aged 9–15 years during the study period. In addition, all quartiles showed an apparent U‐shaped pattern, with the lowest FEV_1_/FVC ratio observed around the age of 11 years in males; however, this pattern was observed only in one quartile group in females.

The trajectory of lung function growth in children around puberty was extensively studied decades ago; however, almost all studies were retrospective in nature and based on data from Western and African‐American participants (Borsboom et al., 1996; Lebowitz & Sherrill, 1995; Sherrill et al., 1989; Wang et al., 1993). To the best of our knowledge, this is the first study to examine the peripubertal lung growth pattern through a prospective longitudinal follow‐up of the same cohort of East Asian children. Several factors may influence the trajectories of PFTs in relation to ethnicity. Genetic and lifestyle factors, including dietary habits and/or exercise, may influence lung function growth patterns. In general, the mean height of Japanese adults is lower than that of their Western and African‐American counterparts. As lung function is approximately linked to height, we hypothesized that the growth pattern of lung function might differ from that of other ethnic groups. Thus, we wondered whether the growth pattern of lung function might differ from that of other ethnic groups.

We found that the later the onset of height growth spurt, the greater the lag between APHV and lung function variables, contradictory to the finding of Wang et al. (1993). They demonstrated that the length of the lag period between height and FEV_1_ and FVC was 0.6–0.9 years, on average, and emphasized that the earlier the height growth spurt, the greater the lag in peak velocity between height and lung function growth. The exact reasons for this discrepancy are unclear; however, plausible explanations may be as follows: first, they only analyzed children with identifiable lung growth spurt retrospectively, thus excluding a substantial number of children; second, unlike our SITAR model, they did not apply any modern mixed‐effects models that account for within‐subject correlation in repeated measures for estimating peak growth velocity. However, direct comparisons of findings between their report and ours may be difficult because of the different ethnic populations encountered. Nonetheless, we believe that our observation is universally true and more likely accounts for the background phenomenon reported by Mahmoud et al., wherein they demonstrated that the later APHV is significantly associated with maximal attained lung function at 24 years of age in both sexes (Guerra et al., 2004).

The FEV_1_/FVC ratio provides a crude index of airway size normalized by lung size in healthy individuals; thus, it is a parameter indicating parenchymal airway dysanapsis (i.e., unequal growth), as first defined by Mead (1980). The FEV_1_/FVC ratio in male children is lower than that in female children (Wang et al., 1993); this sex‐related difference in FEV_1_/FVC values may be extrapolated to adulthood, as females have a higher FEV_1_/FVC ratio than males throughout life, at least in the Japanese population (Kubota et al., 2014; Lung physiology expert committee of Japanese Respiratory Society, 1993). Several epidemiological surveys suggest that lower FEV_1_/FVC ratios in male children than in female children may account for the higher susceptibility of males to the effects of passive smoking on lung function (Lebowitz et al., 1992; Murray & Morrison, 1989). Moreover, the prevalence and severity of cystic fibrosis (Harness‐Brumley et al., 2014), and the sex differences in elite swimming performance in youth may also be explained by this phenomenon (Senefeld et al., 2019).

However, this sex‐related difference in the prevalence of some airway diseases is generally reversed in adults, particularly in those with asthma (Akinbami et al., 2012; Leynaert et al., 2012). Of note, a recent Japanese national survey on the prevalence of allergic diseases demonstrated that the prevalence of wheeze was more predominant in male children than in female children until 10 years of age; however, this phenomenon is reversed during later years (Ito et al., 2023). Regarding the trajectory of the FEV_1_/FVC ratio during childhood, one study demonstrated a U‐shaped curve, with the lowest values observed at approximately 12 years in male and 10 years in female children (Wang et al., 1993). In the present study, while a U‐shaped phenomenon was observed in male children with the lowest FEV_1_/FVC ratio around the age of 11–12 years in all quartiles, in female children, this was observed only in one quartile. Consequently, it seems that male children catch up with female children in FEV_1_/FVC values after puberty; a closer look at the absolute values of FVC and FEV_1_ highlights that male children have higher values of both after puberty, compared with those in females.

An absolute value of FEV_1_, rather than FEV_1_/FVC, is assumed to reflect airway size; sex‐related differences in the trajectory of FEV_1_ as well as FEV_1_/FVC ratio around puberty may partially explain the epidemiological phenomenon of sex‐related asthma prevalence (Akinbami et al., 2012; Ito et al., 2023; Leynaert et al., 2012). It is noteworthy that respiratory function variables, if expressed as a function of height (not of age), are almost precisely aligned, regardless of quartile based on APHV (Figure 6 and Figure S4) in both sexes. These data clearly suggest that respiratory function growth is basically dependent on height growth even though there is an apparent difference in the timing of peak growth velocity between the two. Furthermore, sex‐related differences were evident in absolute values of FVC and FEV_1_ when height exceeded 150–160 cm. This height likely corresponds to the timing of puberty and indicates that lung size, including both airways and parenchyma, will become larger after puberty in male children than in female children, even if they are matched by height.

There are certain limitations to this study. First, the observation period was limited, that is, children were followed up until the age of 15 years. Height continues to increase after this age, particularly in male children, and lung growth continues even after the growth spurt in height has stopped (Mahmoud et al., 2018). To overcome this limitation, we classified the children quarterly according to APHV, allowing the depiction and identification of the peak lung function growth velocity in most, but not all, quartiles. Nonetheless, we could not identify both APHV in Q4 and peak growth velocity of lung function variables in Q3–Q4 among male children, mainly because of the limited observation period between 9 and 15 years of age. Second, for the analysis of dysanapsis, we should have used the small airway parameter, such as V50 or/and FEF20–75%, instead of FEV_1_ corrected by lung size. However, as shown in Appendix S1, the variable V50 showed a considerably wide intraindividual variation, with the data not reliable enough to draw a definite conclusion. Next, the number of participants at the end of the study reduced to almost 60% of the original participants because some junior high schools did not accept our study proposal. We performed analyses for the available data, whereby we included only cases with data for each visit on the variables of interest, without imputing missing values. However, the primary reason for missing data was not declines in lung function or related problems. Indeed, as shown in Table S1, there were no significant differences in the measurements across school grades based on whether further follow‐up was possible or not. Therefore, we believe that the impact of missing data on the results is minimal. Using well‐established sophisticated statistical approaches, we could successfully analyze the whole data throughout the study period. Finally, this study was exploratory in nature and was not designed with an a priori hypothesis or power calculations. However, the study included a large sample, which strengthens the interpretation and generalizability of the findings.

In conclusion, we successfully depicted the trajectory of lung function growth pattern in relation to APHV in Japanese children aged 9–15 years. The data indicate significant variabilities of lung growth pattern dependent on APHV and demonstrate sex‐related differences in the lung growth of airways and parenchymal components. This study offers clinicians and researchers new insights into the interpretation of lung function measurements around puberty and potentially for the future adolescent period.

## AUTHOR CONTRIBUTIONS

Masaharu Nishimura, Satoshi Konno, and Masataka Taguri designed the study, analyzed the data, and wrote the manuscript. Hiroshi Odajima, Mihoko Minami, Toru Takebayashi, and Hiroshi Nitta designed the study, obtained data sets, and contributed to the manuscript.

## FUNDING INFORMATION

Supported by the Ethical Committee of the Ministry of the Environment of Japan (approval number 11021001). The findings and conclusions of this article are solely the responsibility of the authors and do not represent the official views of the above government.

## ETHICS STATEMENT

The study was centrally approved by the Ethical Committee of the Ministry of the Environment of Japan (Approval number: 11021001) and also by each regional study center.

## Supporting information


Appendix S1.
