# Supplementary material for: Peripubertal lung growth pattern in Japanese school children
Source: Physiol Rep. 2025 Aug 26;13(16):e70508. doi: 10.14814/phy2.70508 (PMC12381360; doi:10.14814/phy2.70508)
Supplement: Supplementary file 1 — Appendix S1. [file PHY2-13-e70508-s001.docx]

Supplemental file

**Lung growth pattern around puberty in Japanese school children**

Satoshi Konno MD ^1)^ *, Masataka Taguri PhD ^2)^ *, Hiroshi Odajima MD ^3)^ , Mihoko Minami PhD ^4)^,Toru Takebayashi MD ^5),^ Hiroshi Nitta PhD ^6)^, Masaharu Nishimura MD ^1)7)^

*, equally contributed to this work

Pulmonary Function Tests

PFTs for the children in the third to sixth grades were scheduled during the same season every year to minimize seasonal effects such as those of temperature and pollution levels; the same spirometers with Lilly-type pneumotach sensors (Chest HI801, CHEST M.I., Inc., Tokyo, Japan) were used for testing at all locations. In all schools except two, sixth-grade children underwent an additional PFT in February or March (i.e., a month before they moved to junior high school). In each school the time of performing PFT was different. But, PFT was conducted at almost the same time throughout the study in each school. Each time, children’s heights and weights were measured right before the PFT. Height was measured to the nearest millimeter, and weight was measured to the nearest 100 milligrams.

When conducting PFT for children, we implemented various strategies. First, we used picture-story which shows to demonstrate how the test is performed. After that, we had a subject actually perform the test in front of the other children, aiming to make them perceive it as a fun experience. During the actual testing, we never scolded them for mistakes, instead, we praised what they did well. Even when they made mistakes, we provided a one-minute break at least after each manuver, and during this time, we engaged them in conversation to prevent boredom.

The tests were conducted by six trained technicians following the testing protocol of the ATS/ERS standards (1). Maximum forced expiratory maneuver was performed in standing.

We limited the number of test attempts to a maximum by no more than 6 repeated maneuvers in each test. The FEV_1_, FVC, and maximal expiratory flow rate at 50% of FVC (V50) were determined from three satisfactory blows delivered under the guidance of two pediatric pulmonologists.

**Reference**

1 Miller MR, Hankinson J, Brusasco V, Burgos F, Casaburi R, Coates A, et al. Standardisation of spirometry. Eur Respir J 2005;26:319-338. doi: [10.1183/09031936.05.00034805](https://doi.org/10.1183/09031936.05.00034805).

Supplement Table S1. Differences between the two groups (children we could/could not follow up)

| **Sex** | **Variable** | **Participants whom we could follow up** | | | | | | | **Participants whom we could not follow up** | | | | | **Comparison between two groups ***  **(p-value)** | | | | |
| --- | --- | --- | --- | --- | --- | --- | --- | --- | --- | --- | --- | --- | --- | --- | --- | --- | --- | --- |
|  |  | **3^rd^** | **4^th^** | **5^th^** | **6^th^(1)** | **6^th^(2)** | **8^th^** | **9^th^** | **3^rd^** | **4^th^** | **5^th^** | **6^th^(1)** | **6^th^(2)** | **3^rd^** | **4^th^** | **5^th^** | **6^th^(1)** | **6^th^(2)** |
| **Male** | **Number of participants** | 410 | 466 | 463 | 460 | 405 | 372 | 372 | 199 | 203 | 208 | 193 | 182 | **－** | **－** | **－** | **－** | **－** |
|  | **Age (year)** [mean(SD)] | 8.80(0.322) | 9.78(0.332) | 10.78(0.333) | 11.78(0.336) | 12.36(0.297) | 13.84(0.363) | 14.83(0.363) | 8.86(0.299) | 9.84(0.300) | 10.84(0.307) | 11.83(0.308) | 12.41(0.291) | **0.050** | **0.027** | **0.033** | **0.048** | 0.095 |
|  | **Height (cm)** [mean(SD)] | 129.7(5.56) | 135.1(6.02) | 140.8(6.49) | 147.4(7.66) | 151.6(8.00) | 162.1(7.18) | 166.6(6.05) | 130.3(5.71) | 135.6(6.07) | 141.3(6.54) | 147.5(7.42) | 151.8(7.86) | 0.231 | 0.309 | 0.383 | 0.869 | 0.845 |
|  | **Weight (kg)** [mean(SD)] | 27.4(5.27) | 30.8(6.13) | 34.6(7.26) | 39.2(8.54) | 42.3(8.93) | 50.5(9.61) | 54.7(9.01) | 27.7(6.04) | 31.1(7.16) | 34.8(8.08) | 38.8(8.67) | 42.5(9.10) | 0.644 | 0.642 | 0.761 | 0.602 | 0.790 |
|  | **FVC (L)**  [mean(SD)] | 1.91(0.268) | 2.11(0.308) | 2.34(0.352) | 2.64(0.457) | 2.89(0.525) | 3.55(0.596) | 3.93(0.575) | 1.94(0.269) | 2.11(0.316) | 2.39(0.353) | 2.67(0.437) | 2.95(0.502) | 0.137 | 0.915 | 0.145 | 0.548 | 0.179 |
|  | **FEV_1_ (L)**  [mean(SD)] | 1.68(0.234) | 1.85(0.267) | 2.03(0.299) | 2.29(0.394) | 2.50(0.463) | 3.14(0.551) | 3.47(0.531) | 1.71(0.229) | 1.84(0.260) | 2.07(0.294) | 2.31(0.356) | 2.55(0.448) | 0.083 | 0.898 | 0.192 | 0.605 | 0.233 |
|  | **V_50_ (L/s)**  [mean(SD)] | 2.37(0.549) | 2.57(0.590) | 2.77(0.646) | 3.10(0.758) | 3.33(0.849) | 4.14(1.052) | 4.55(1.057) | 2.41(0.533) | 2.51(0.581) | 2.77(0.605) | 3.02(0.669) | 3.36(0.880) | 0.385 | 0.191 | 0.955 | 0.180 | 0.698 |
| **Female** | **Number of participants** | 408 | 470 | 466 | 447 | 382 | 366 | 361 | 174 | 182 | 182 | 181 | 154 | **－** | **－** | **－** | **－** | **－** |
|  | **Age (year)** [mean(SD)] | 8.82(0.327) | 9.82(0.336) | 10.81(0.338) | 11.81(0.346) | 12.39(0.291) | 13.87(0.375) | 14.85(0.366) | 8.80(0.318) | 9.80(0.315) | 10.81(0.311) | 11.79(0.311) | 12.40(0.297) | 0.596 | 0.444 | 0.876 | 0.647 | 0.521 |
|  | **Height (cm)** [mean(SD)] | 129.0(5.48) | 135.3(6.38) | 142.3(6.95) | 148.7(6.68) | 151.6(6.00) | 155.7(5.10) | 156.9(5.11) | 129.4(5.96) | 135.5(6.63) | 142.2(7.48) | 148.3(7.13) | 151.7(6.35) | 0.385 | 0.657 | 0.984 | 0.565 | 0.885 |
|  | **Weight (kg)** [mean(SD)] | 26.6(4.85) | 30.1(5.71) | 34.5(6.91) | 39.5(7.80) | 42.3(7.67) | 48.0(8.24) | 50.3(8.04) | 27.5(5.65) | 31.0(6.55) | 35.4(7.69) | 40.2(8.26) | 44.3(8.53) | 0.074 | 0.114 | 0.183 | 0.348 | **0.014** |
|  | **FVC (L)**  [mean(SD)] | 1.75(0.251) | 1.94(0.290) | 2.21(0.357) | 2.50(0.395) | 2.69(0.416) | 2.96(0.395) | 3.04(0.410) | 1.74(0.244) | 1.96(0.283) | 2.26(0.367) | 2.51(0.427) | 2.71(0.420) | 0.881 | 0.609 | 0.216 | 0.788 | 0.666 |
|  | **FEV_1_ (L)**  [mean(SD)] | 1.58(0.226) | 1.76(0.261) | 2.00(0.328) | 2.27(0.364) | 2.44(0.384) | 2.69(0.352) | 2.75(0.375) | 1.58(0.216) | 1.77(0.261) | 2.02(0.329) | 2.27(0.381) | 2.45(0.369) | 0.749 | 0.634 | 0.675 | 0.879 | 0.727 |
|  | **V_50_ (L/s)**  [mean(SD)] | 2.44(0.543) | 2.69(0.586) | 3.03(0.743) | 3.44(0.769) | 3.59(0.829) | 3.91(0.818) | 4.02(0.853) | 2.44(0.513) | 2.71(0.614) | 3.06(0.732) | 3.40(0.808) | 3.66(0.823) | 0.931 | 0.627 | 0.632 | 0.593 | 0.385 |

SD= standard deviation.

* Comparison was performed (participants living in the cities that we could and could not follow up) using *t*-test

Figure S1.


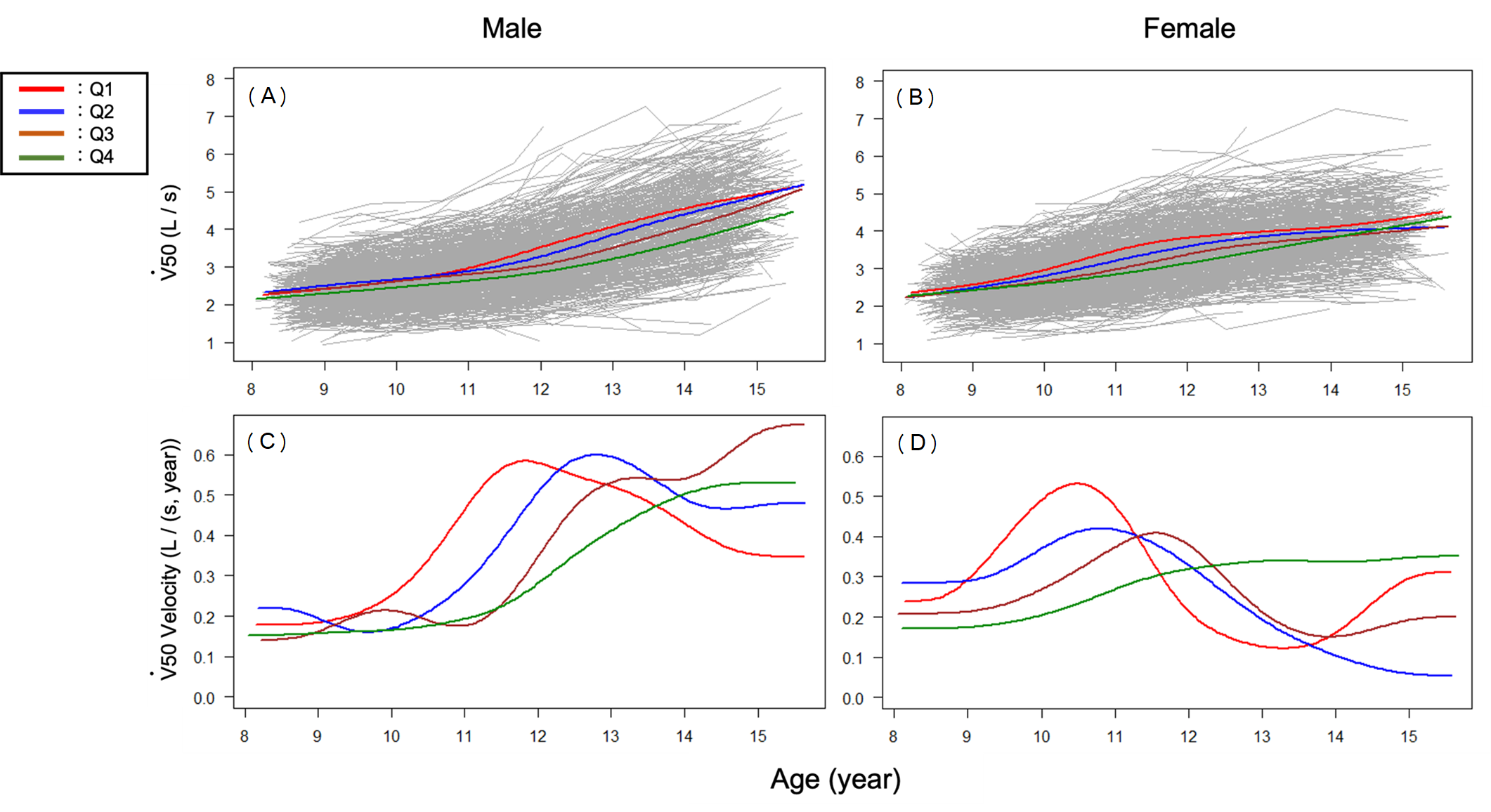


Figure S2

Figure S3

Figure S4

Figure S5


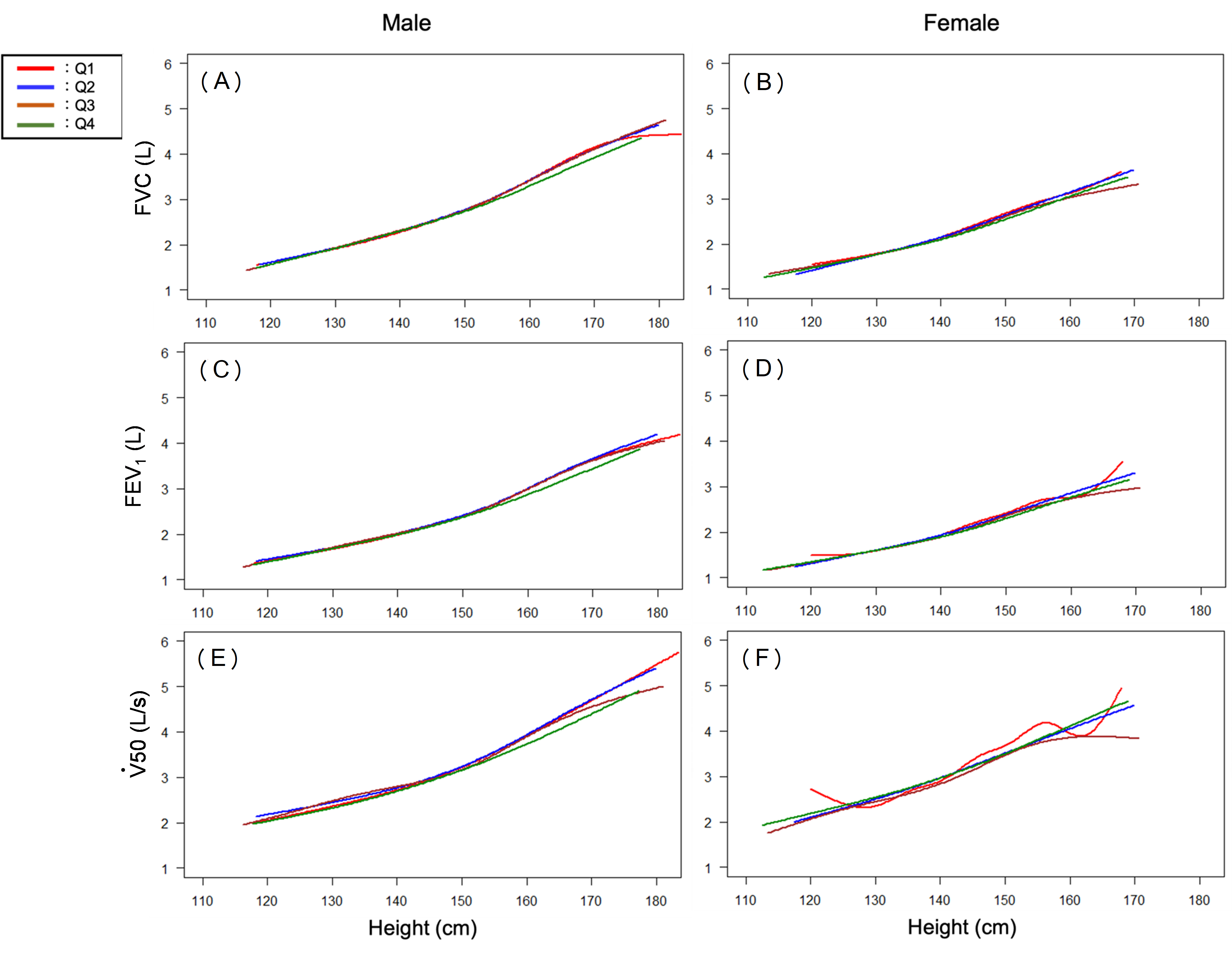


Figure S6

**Supplemental Figure legends**

**Figure S1.** Histogram of the estimated age at peak height velocity (APHV) using the superimposition by translation and rotation (SITAR) model. The left panels are for male children, and the right panels are for female children. Red lines indicate the estimated APHV quartiles.

**Figure S2.** Growth curve and velocity for 50 for male children in Q1 to Q4, which represent quartiles based on the age at peak height velocity (APHV). The left panels are for male children, and the right panels are for female children. V50, maximal expiratory flow rate at 50% of FVC

**Figure S3**. Growth velocity for height, FVC, FEV_1_, and V50 for male children in Q1–Q4, which represent quartiles based on the age at peak height velocity (APHV). The number in the top left corner of each figure indicates the peak age. The two red vertical lines represent the 10th and 90th percentiles of age at the time of measurement, respectively. The solid blue line represents the estimated growth velocity, and the blue dashed line represents the 95% confidence interval. "NE" represents “not estimable.”

FEV_1_, forced expiratory volume in the first one second; FVC, forced vital capacity

**Figure S4**. Growth velocity for height, FVC, FEV_1_, and V50 for female children in Q1–Q4, which represent quartiles based on the age at peak height velocity (APHV). The number in the top left corner of each figure indicates the peak age. The two red vertical lines represent the 10th and 90th percentiles of age at the time of measurement, respectively. The solid blue line represents the estimated growth velocity, and the blue dashed line represents the 95% confidence interval. "NE" represents “not estimable.”

**Figure S5.** Peak age differences to height and their 95% confidence intervals for FEV_1_ and FVC in Q1 to Q4, which represent quartiles based on the age at peak height velocity (APHV). FEV_1_, forced expiratory volume in the first one second; FVC, forced vital capacity

**Figure S6.** Pulmonary function variables drawn as a function of height instead of age. The four lines were almost identical in both sexes, regardless of quartile. FEV_1_, forced expiratory volume in the first one second; FVC, forced vital capacity, V50, maximal expiratory flow rate at 50% of FVC
